# Supplementary material for: Auto-qPCR; a python-based web app for automated and reproducible analysis of qPCR data
Source: Sci Rep. 2021 Oct 29;11:21293. doi: 10.1038/s41598-021-99727-6 (PMC8556264; doi:10.1038/s41598-021-99727-6)
Supplement: Supplementary file 1 — Supplementary Information. [file 41598_2021_99727_MOESM1_ESM.pdf]

Supplementary Figure Legends

Supplementary Figure S1. Screen shot of Auto-qPCR interface.

Supplementary Figure S2. Examples of results spreadsheet files to use as input for Auto-qPCR. (**A**) Spreadsheet with column names needed. (**B**) Screen shot of the top of the csv saved from the results sheet of the exported Excel file. (**C**) Screen shot of the column names in the save results file that will be read into Auto-qPCR.

Supplementary Figure S3. Example output from Auto-qPCR using the genomic instability model. (**A**) The Log.txt output from the file generated by Auto-qPCR. The file lists the steps completed by the program and the inputs from the web interface. This example is from the genomic instability analysis. The selection for statistical analysis is also shown in the text file. Using the log file, the exact analysis can be repeated because all the settings are recorded. (**B**) Bar chart showing an alternative visualization for the genomic instability assay where the data is grouped by cell lines on the x-axis and colours indicated in the legend represent the regions of chromosomes tested.

Supplementary Figure S4. Screen shots of options entered into Auto-qPCR web app to analyze the example data for the absolute model in Fig. 3. (**A**) Options to produce the summary data. (**B**) Statistics options.

Supplementary Figure S5. Example images of AJG001-C4 at four stages of development (iPSCs, NPCs, as well as 4 and 6 week DANs). (**A**) iPSCs stained for pluripotency markers (Nanog, Tra1-60, SSEA4, OCT3-4 as indicated), together with Hoechst and shown as merged images on the right. (**B**) Neural precursor cells (NPCs) expressing dopaminergic lineage (SOX1 and OTX2), proliferation (Ki67) and neural progenitors (Nestin) markers. (**C**) Dopaminergic neurons after 4 and 6 weeks of differentiation stained with neuronal marker Tuj1 in all images and dopaminergic markers FOXA2, GIRK2 and TH as indicated.

Supplementary Figure S6. Screen shots of options entered into Auto-qPCR web app to analyze the example data for the relative models in Fig. 4. (**A**) Options to produce the summary data using the relative ΔCT method, where values are normalized to the endogenous controls (ACTB and GAPDH). (**B**) Options to produce the

summary data using the relative ΔΔCT method, where expression values are normalized both the endogenous controls and the reference sample.

Supplementary Figure S7. Screen shot of options entered into Auto-qPCR web app to for statistical analysis in Fig. 4 using relative models. Statistics options used, the selections are the same for both the ΔCT and the ΔΔCT normalization methods.

Supplementary Figure S8. Screen shot of options entered into Auto-qPCR web app to for analysis of the input used for the absolute quantification to reprocess data from the Opticon 2 Biorad thermocycler. (**A**) Screen shot of file names that contain the endogenous control and the gene to be analyzed. (**B**) Screen shot of Auto-qPCR with the file names and entered under file information. All the options entered are to create the summary data used in **Fig. 5**.

Supplementary Figure S9. Screen shot of options entered for statistical analysis into Auto-qPCR web app for the absolute quantification to reprocess data from the Opticon 2 Biorad thermocycler. (**A**) Statistics options to compare brain regions and treatment combined to create 6 groups, a one-way ANOVA will be performed. (**B**) Statistic options to compare treatment and control (the brain regions are treated as one group), a t-test will be performed.

Supplementary Figure S10. Screen shot of options entered for statistical analysis into Auto-qPCR web app for the absolute quantification to reprocess data from the Opticon 2 Biorad thermocycler. (**A**) Statistics options to compare brain regions with control vs. cocaine treated as one group, a one-way ANOVA will be performed with three brain regions as groups. (**B**) Statistic options for the two-way ANOVA where interaction between treatment and control is tested, the two variables are treatment and region.

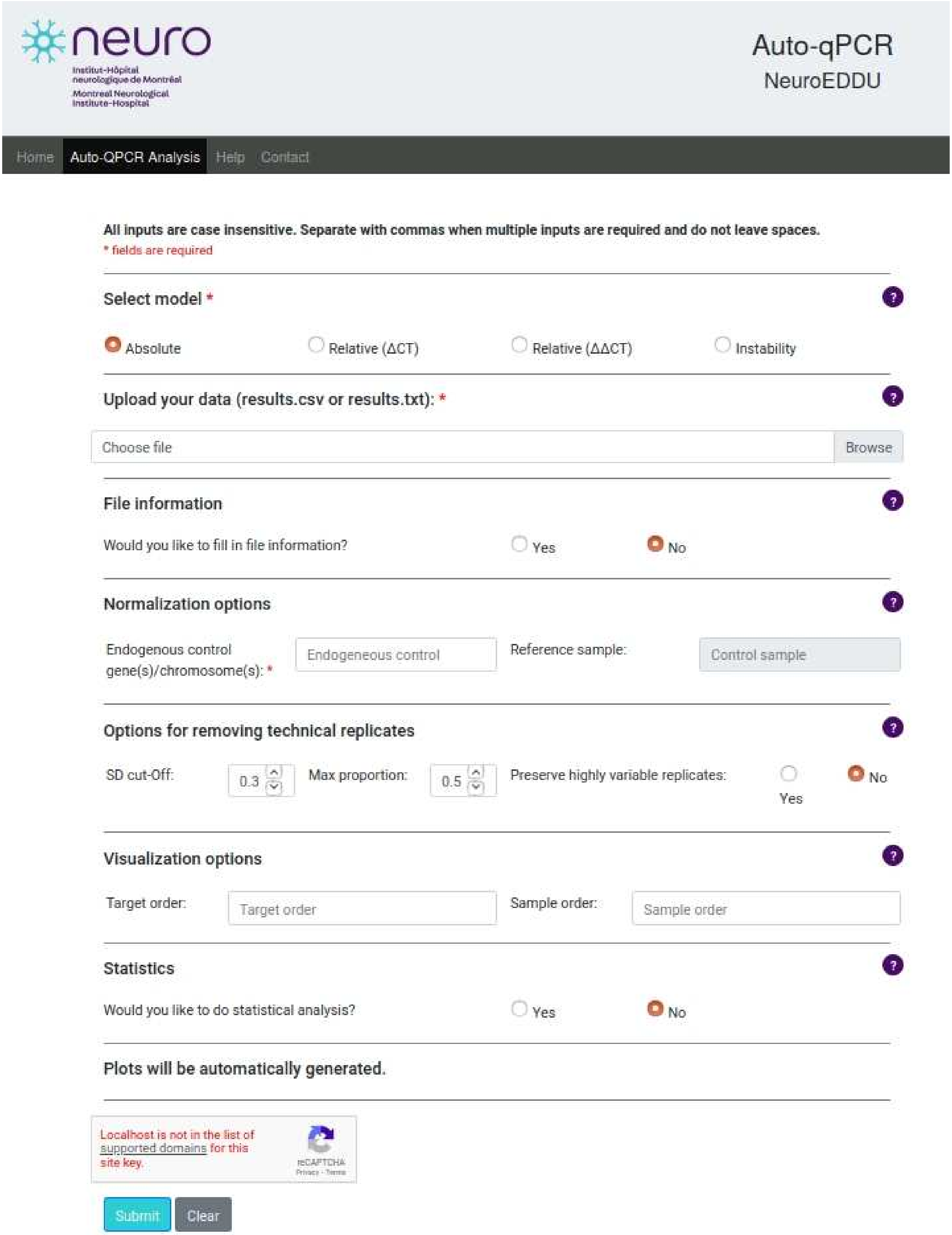

Maussion, Thomas et al. Supplementary Figure S1

A

Column Name	Well	Well Position	Sample Name	Target Name	Task	CT	Quantity	Reporter	Quencher
Example	1	A1	Normal	CHR1	UNKNOWN	24.702		FAM	NFQ-MGB
Column required	Yes	No	Yes	Yes	Yes	Yes	Yes	Yes	No
Values required	No	No	Yes	Yes	Yes	Yes	No	No	No
Purpose	Identify start of columns		Identify samples	Identify gene/target	select rows of data to be included	Input values	For absolute	Can use for filtering	

B

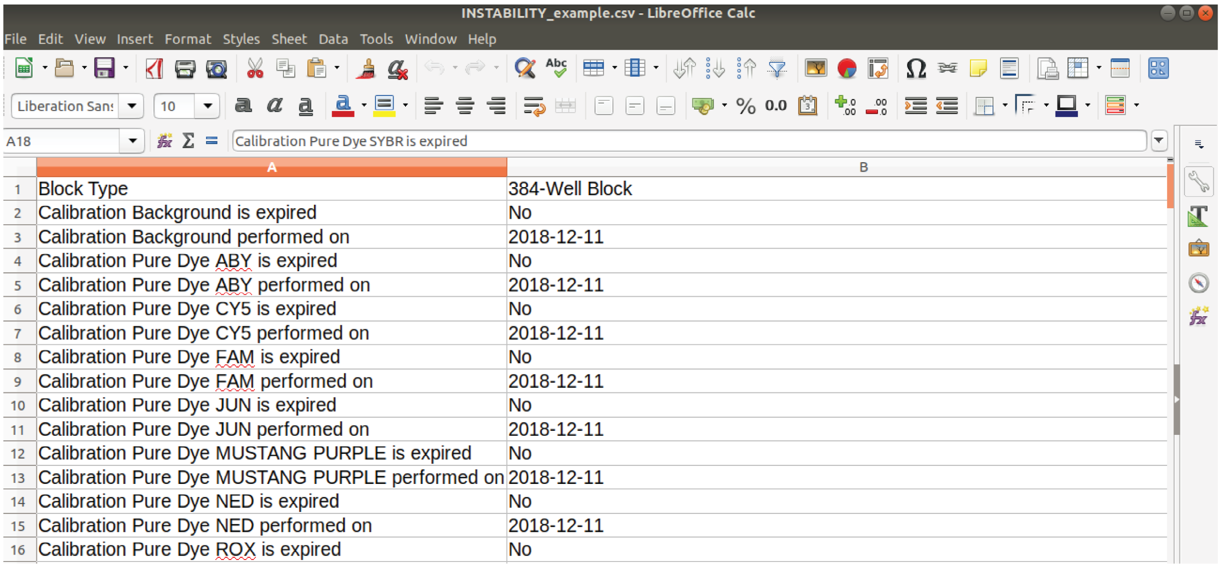
INSTABILITY_example.csv - LibreOffice Calc

File Edit View Insert Format Styles Sheet Data Tools Window Help

A18 | Calibration Pure Dye SYBR is expired

	A	B
1	Block Type	384-Well Block
2	Calibration Background is expired	No
3	Calibration Background performed on	2018-12-11
4	Calibration Pure Dye ABY is expired	No
5	Calibration Pure Dye ABY performed on	2018-12-11
6	Calibration Pure Dye CY5 is expired	No
7	Calibration Pure Dye CY5 performed on	2018-12-11
8	Calibration Pure Dye FAM is expired	No
9	Calibration Pure Dye FAM performed on	2018-12-11
10	Calibration Pure Dye JUN is expired	No
11	Calibration Pure Dye JUN performed on	2018-12-11
12	Calibration Pure Dye MUSTANG PURPLE is expired	No
13	Calibration Pure Dye MUSTANG PURPLE performed on	2018-12-11
14	Calibration Pure Dye NED is expired	No
15	Calibration Pure Dye NED performed on	2018-12-11
16	Calibration Pure Dye ROX is expired	No

C

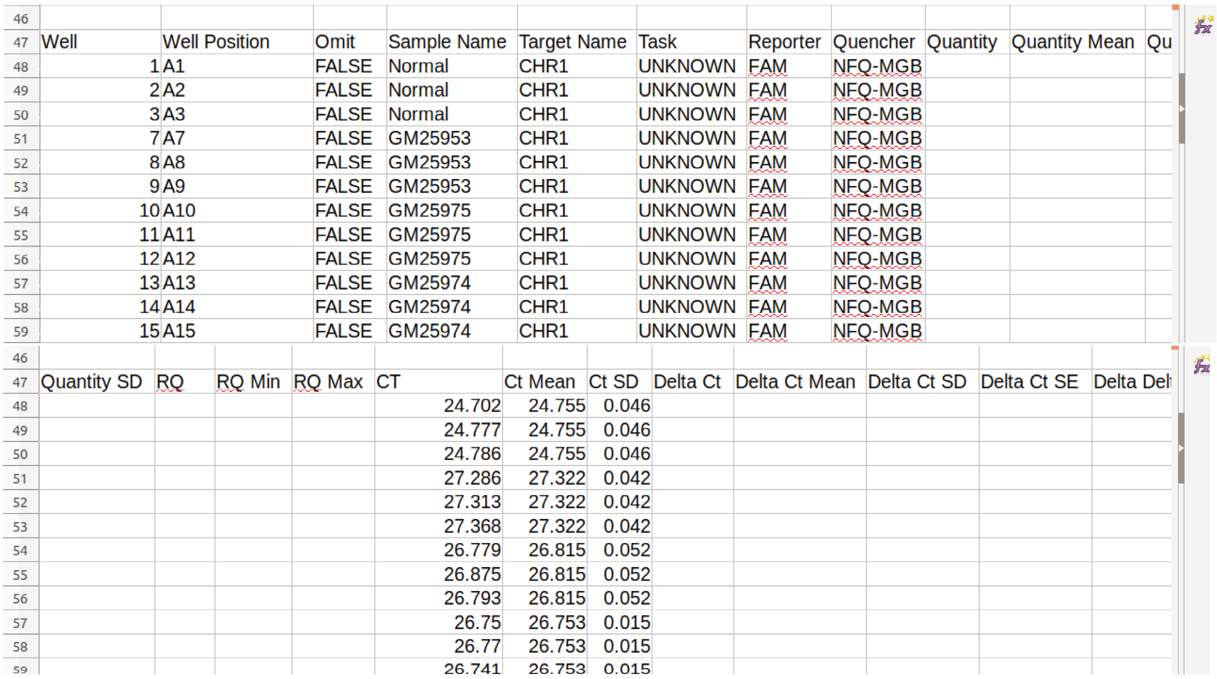

46											
47	Well	Well Position	Omit	Sample Name	Target Name	Task	Reporter	Quencher	Quantity	Quantity Mean	Qu
48	1	A1	FALSE	Normal	CHR1	UNKNOWN	FAM	NFQ-MGB			
49	2	A2	FALSE	Normal	CHR1	UNKNOWN	FAM	NFQ-MGB			
50	3	A3	FALSE	Normal	CHR1	UNKNOWN	FAM	NFQ-MGB			
51	7	A7	FALSE	GM25953	CHR1	UNKNOWN	FAM	NFQ-MGB			
52	8	A8	FALSE	GM25953	CHR1	UNKNOWN	FAM	NFQ-MGB			
53	9	A9	FALSE	GM25953	CHR1	UNKNOWN	FAM	NFQ-MGB			
54	10	A10	FALSE	GM25975	CHR1	UNKNOWN	FAM	NFQ-MGB			
55	11	A11	FALSE	GM25975	CHR1	UNKNOWN	FAM	NFQ-MGB			
56	12	A12	FALSE	GM25975	CHR1	UNKNOWN	FAM	NFQ-MGB			
57	13	A13	FALSE	GM25974	CHR1	UNKNOWN	FAM	NFQ-MGB			
58	14	A14	FALSE	GM25974	CHR1	UNKNOWN	FAM	NFQ-MGB			
59	15	A15	FALSE	GM25974	CHR1	UNKNOWN	FAM	NFQ-MGB			

46												
47	Quantity SD	RQ	RQ Min	RQ Max	CT	Ct Mean	Ct SD	Delta Ct	Delta Ct Mean	Delta Ct SD	Delta Ct SE	Delta Del
48					24.702	24.755	0.046					
49					24.777	24.755	0.046					
50					24.786	24.755	0.046					
51					27.286	27.322	0.042					
52					27.313	27.322	0.042					
53					27.368	27.322	0.042					
54					26.779	26.815	0.052					
55					26.875	26.815	0.052					
56					26.793	26.815	0.052					
57					26.75	26.753	0.015					
58					26.77	26.753	0.015					
59					26.741	26.753	0.015					

Maussion, Thomas et al. Supplementary Figure S2

A

```
Started
Model: instability

Files upload complete.
Gene names if they are included in file names:
Quencher:
Task: UNKNOWN
Endogenous control genes: CHR4
Cut-off: 0.3
Maximum Outliers: 0.5
Preserve highly variable replicates: False
Target Order:
CHR1,CHR4,CHR8,CHR10,CHR12,CHR17,CHR18,CHR20,CHRX
Sample Order: GM25953,GM25975,GM25974,GM25952,Normal
Control Sample: Normal
Additional column names:
Number of groups: None
Group column name:
Group name:
Column name A:
Column Name B:
Group names for column A:
Group names for column B:
Repeated measures: False
Normal distribution: True
Clean data and summary data are created
Plots of the summary data are created.
```

B

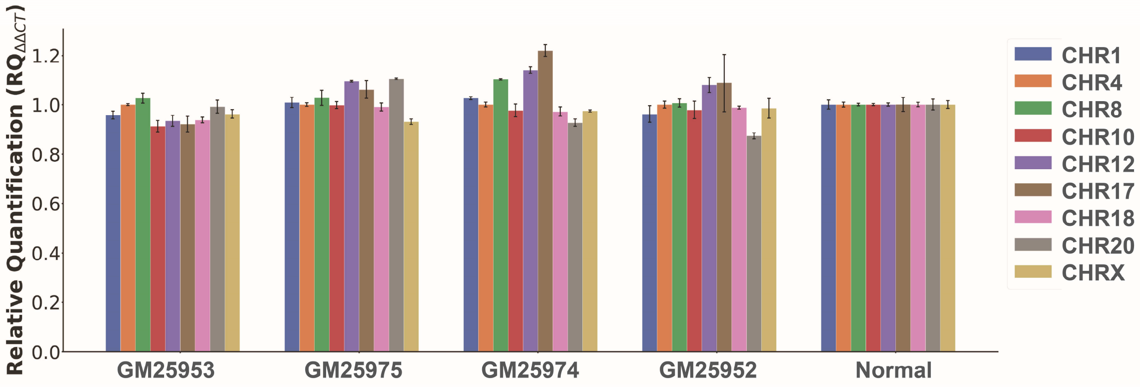

Maussion, Thomas et al. Supplementary Figure S3

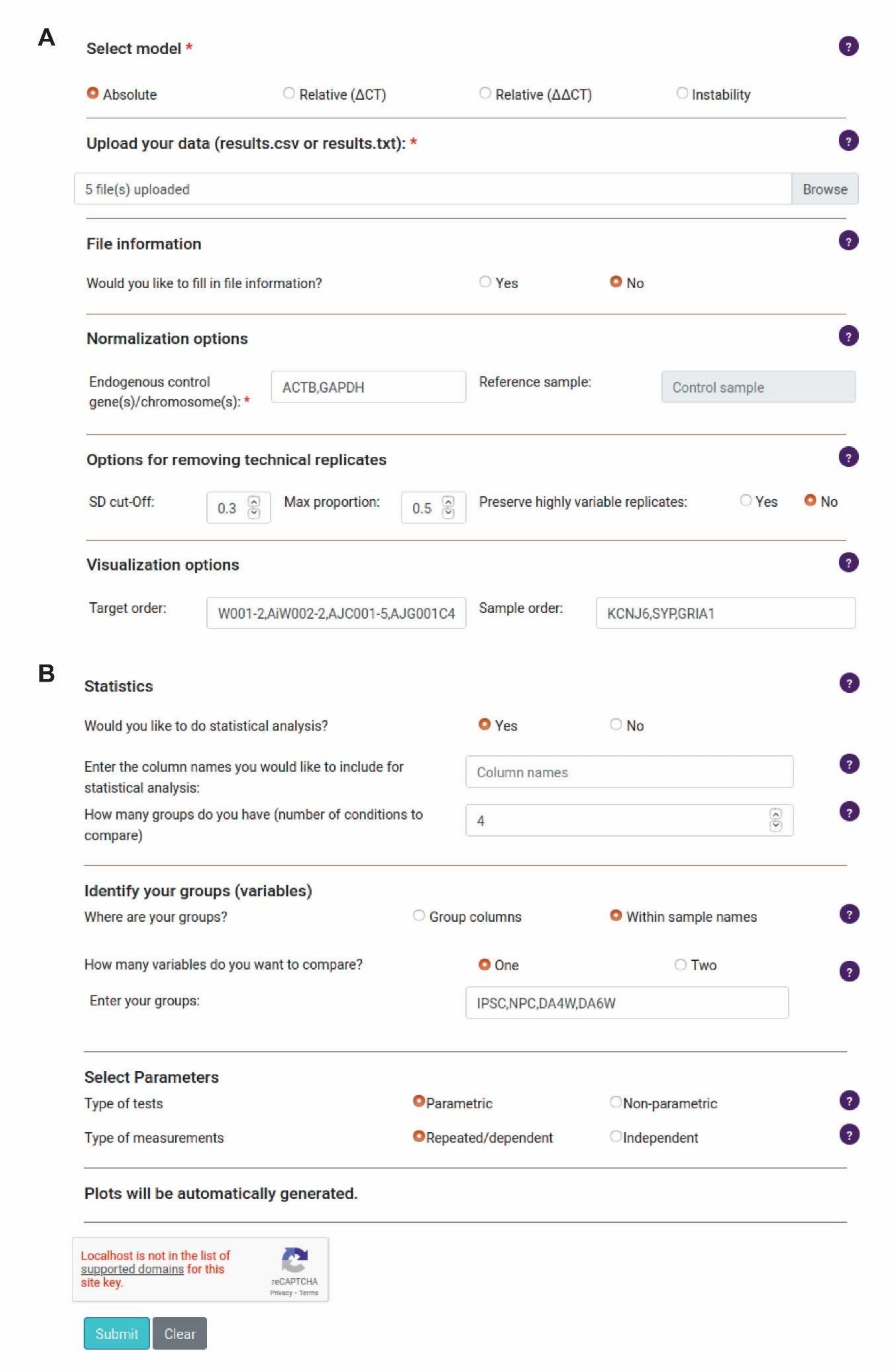

Maussion, Thomas et al. Supplementary Figure S4

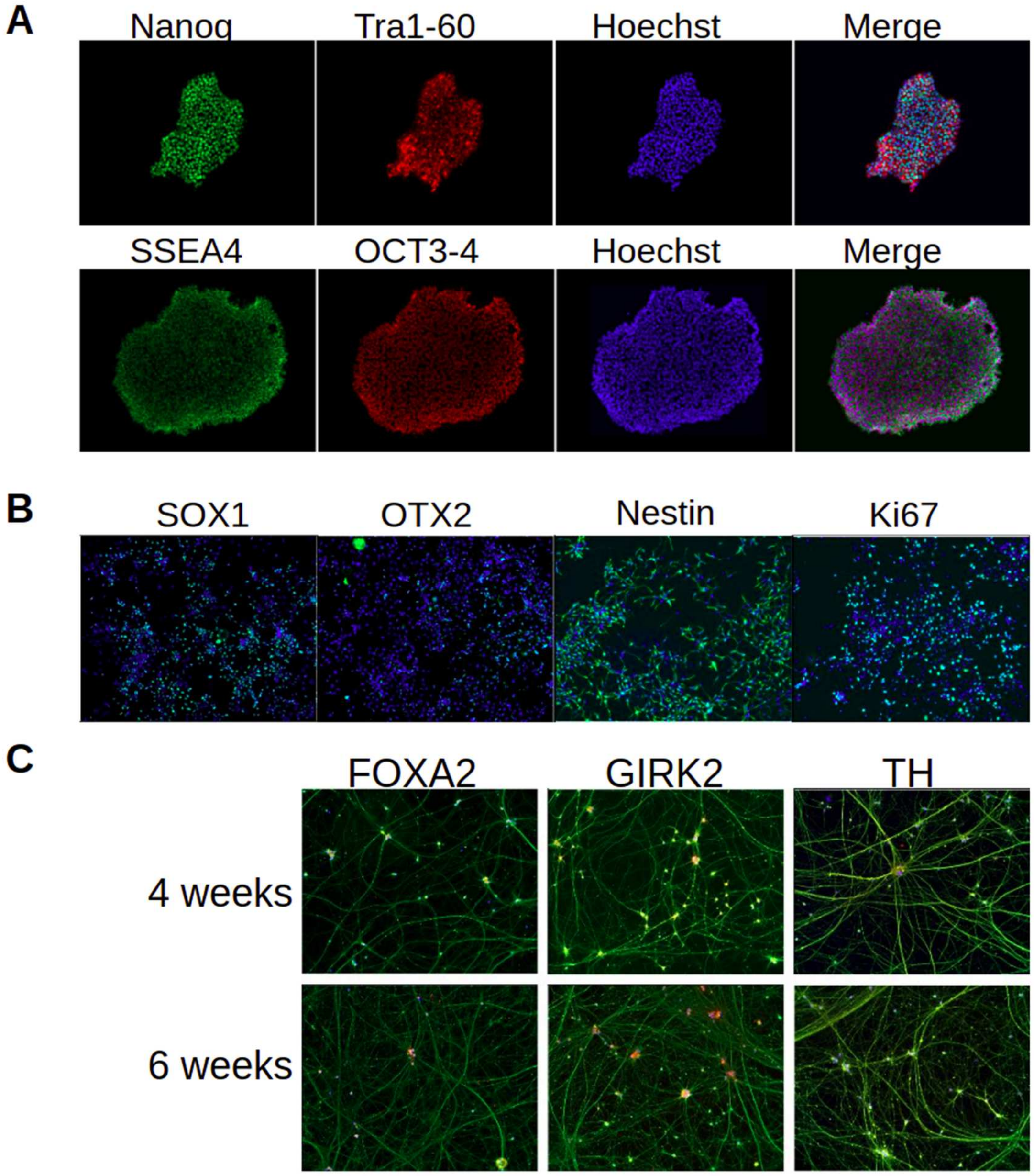

Maussion, Thomas et al. Supplementary Figure S5

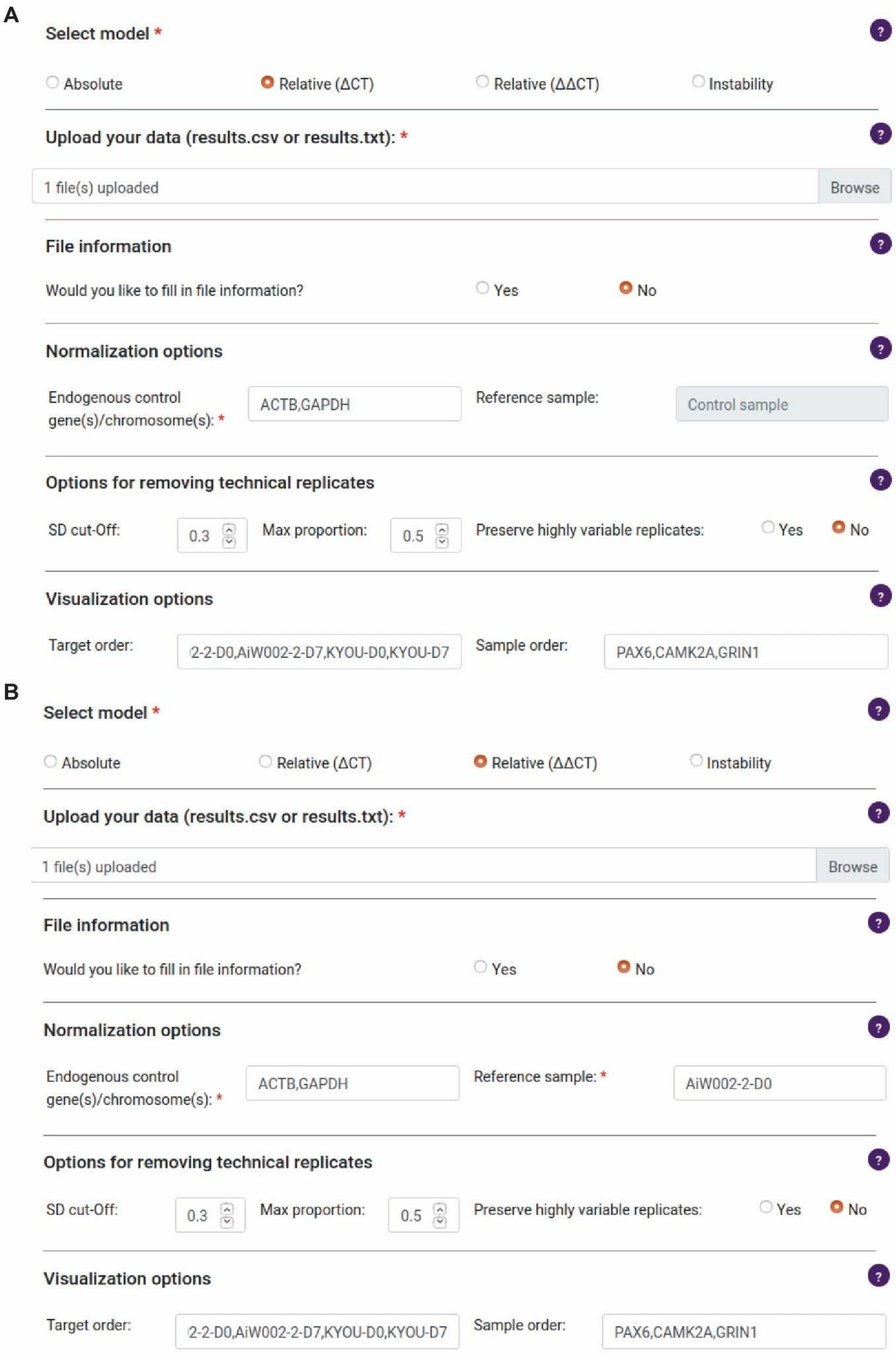

Maussion, Thomas et al. Supplementary Figure S6

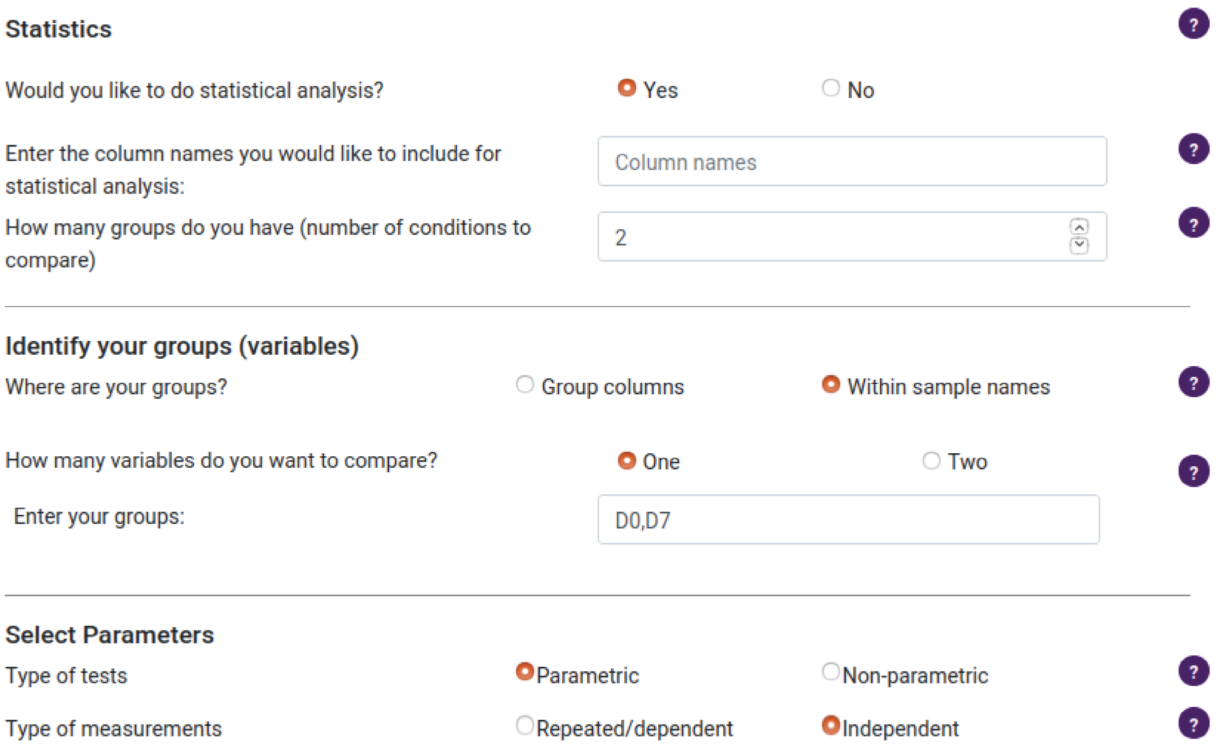

Maussion, Thomas et al. Supplementary Figure S7

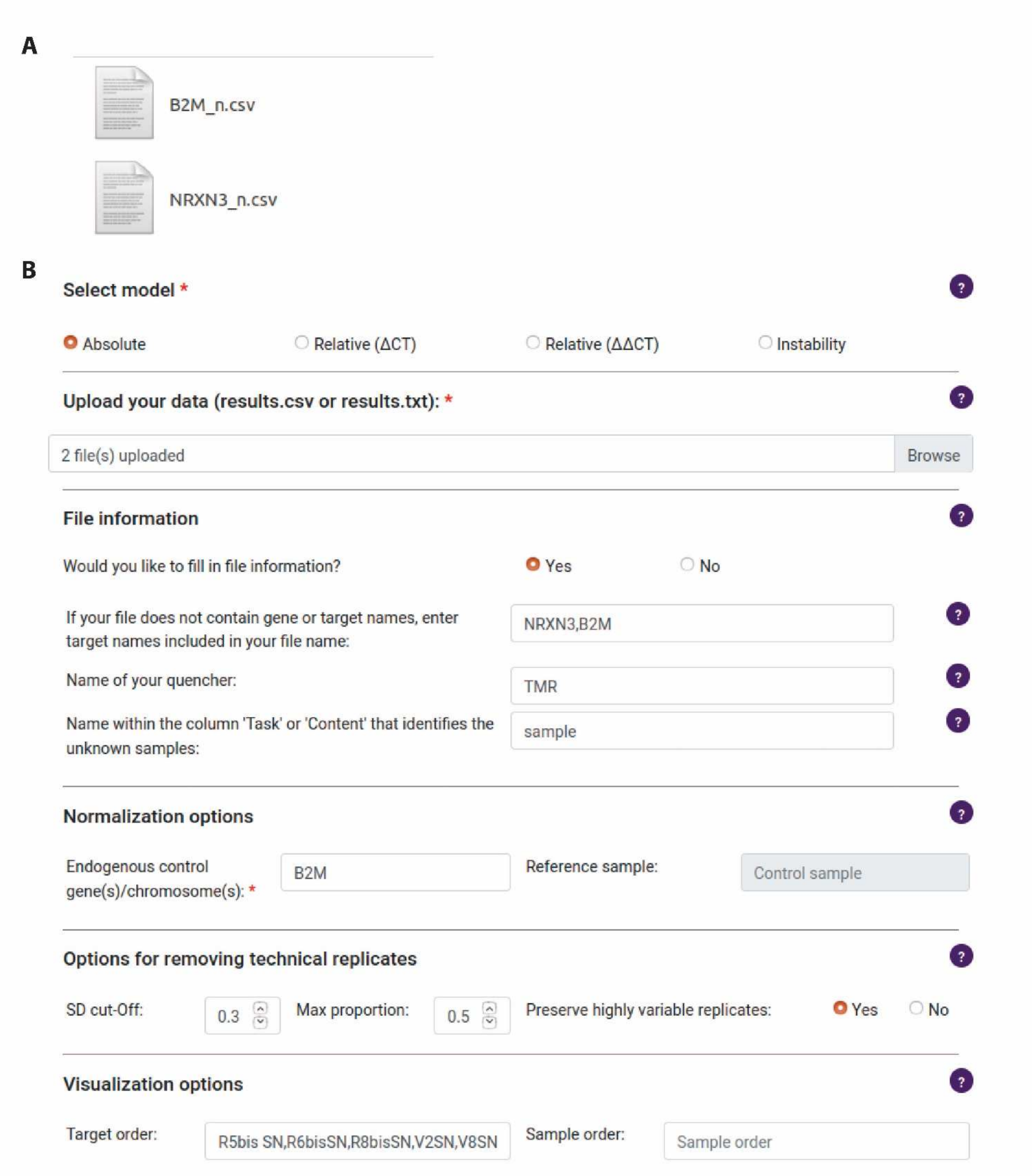

Maussion, Thomas et al. Supplementary Figure S8

A

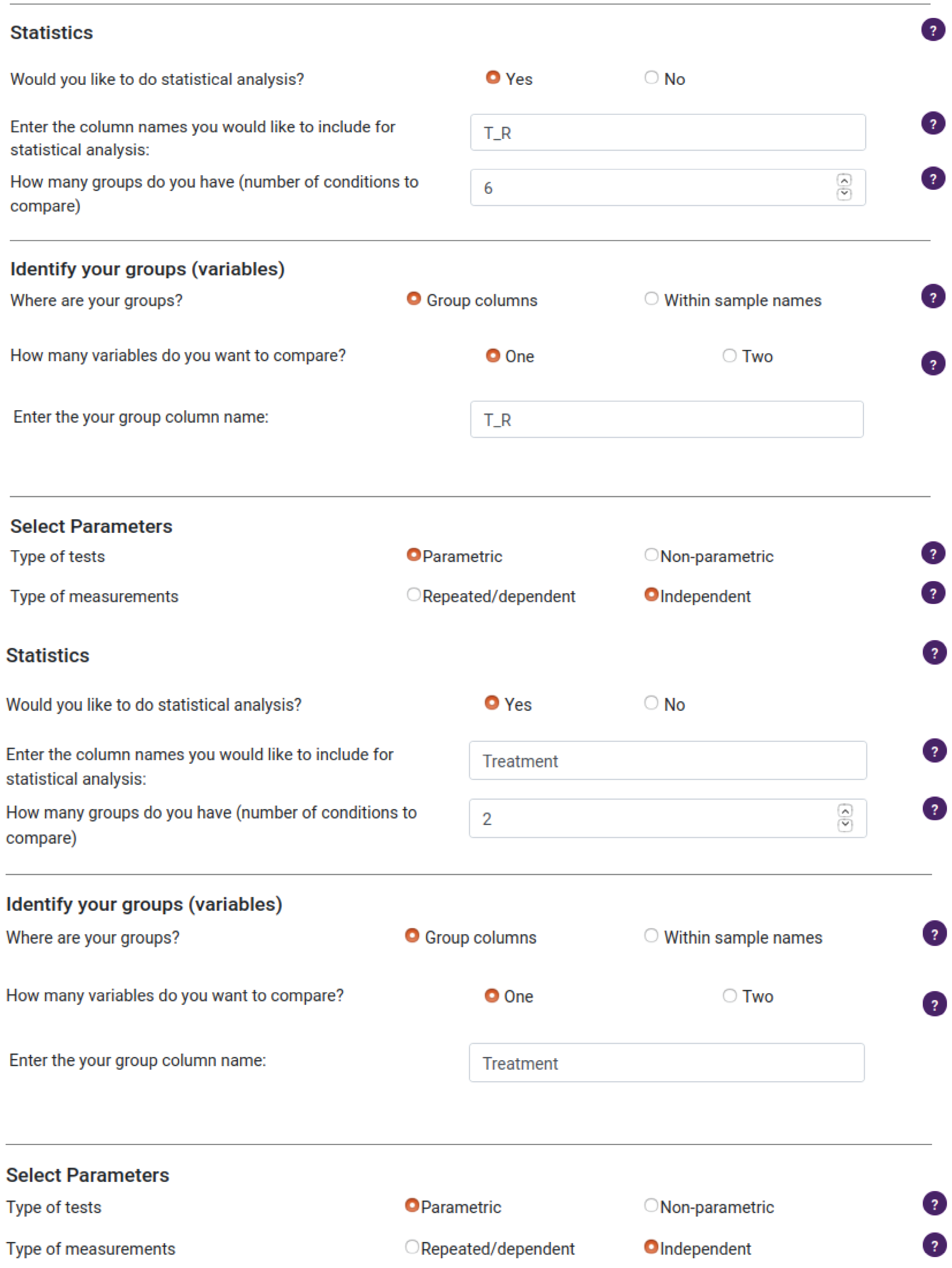

Maussion, Thomas et al. Supplementary Figure S9

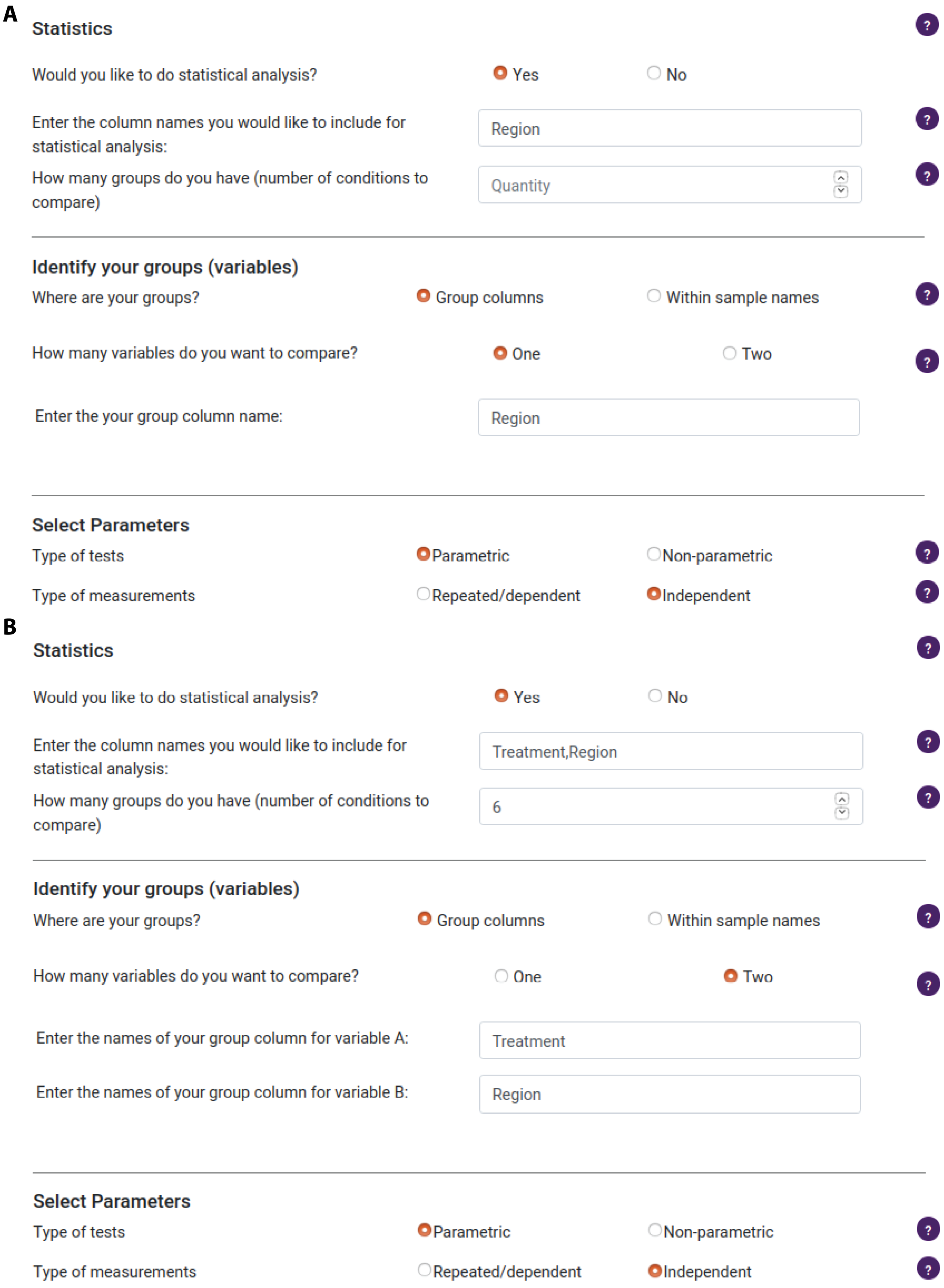

Maussion, Thomas et al. Supplementary Figure S10

Supplemental Tables

Table S1: Overview of cell lines: Human-derived induced pluripotent stem cells used.

Cell line	Donor Age	Sex	Cell Type	Reprogramming Method
GM25952	10	F	Fibroblast	Episomal
GM25953	43	F	Fibroblast	Episomal
GM25974	7	F	Fibroblast	Episomal
GM25975	37	F	Fibroblast	Episomal
522-2666-2	NA	NA	Lymphocytes	Retrovirus
AIW001-2	48	F	PBMCs	Retrovirus
AIW002-2	37	M	PBMCs	Retrovirus
NCRM1	NA	M	Cord Blood	Episomal
AJG001-C4	37	M	PBMCs	Episomal
AJC001-5	37	M	Fibroblast	Retrovirus
KYOU-DRX0190B	36	F	Fibroblast	Retrovirus

Table S2: Taqman primers/probe sets. The primer/probe sets listed were used to generate the data presented in Figures 3 and 4 and test the absolute and relative quantification models to assess gene expression levels by Auto-qPCR web app. The primer/probe sets were selected from the assays available on the Thermo Fisher Scientific web site and chosen to cover the most important number of alternative transcripts for a given gene. With the exception of the assay for GAPDH, the amplicons overlap two exons, avoiding amplification of genomic DNA that could remain from incomplete DNAse digestion. The refseq sequence used for designing the primer/probe set assay is shown.

Gene Symbol	Gene Name	Location	Assay Reference	Exon Boundaries	Reference Accession
ACTB	Actin beta	7p22.1	Hs01060665_g1	2-3	NM_001101
GAPDH	Glyceraldehyde-3-phosphate dehydrogenase	12p13.31	Hs02786624_g1	7	NM_001256799
KCNJ6	Potassium voltage-gated channel subfamily J member 6	21q22.13	Hs01040524_m1	3-4	NM_002240
SYP	Synaptophysin	Xp11.23	Hs00300531_m1	3-4	NM_003179
CAMK2A	Calcium/calmodulin-dependent protein kinase II	5q32	Hs00947041_m1	17-18	NM_015981
PAX6	Paired box 6	11p13	Hs01088114_m1	7-8	NM_000280
GRIN1	Glutamate ionotropic receptor NMDA type subunit 1	9q34.3	Hs00609557_m1	1-2	NM_000832

Table S3: Contents and file structure of Python scripts. The file structure will be maintained if the Auto-qPCR program is downloaded from GitHub and run locally. These files will be found inside the 'website' folder if the GitHub repo is pulled or the zip file is downloaded. Folder Name indicates the parent folder and the subfolder containing the program files. File name indicates the file name for each Python script and Function indicates what processes are performed by each script.

Folder Name	File name	Function
Auto-qPCR	main.py	calls app
application	AUTOqPCR.py	inputs data
		inputs conditions
		removes outliers
		calls model
	absolute.py	runs normalization for absolute model
	relative.py	runs relative quantification with delta-CT normalization
	stability.py	runs relative quantification with delta-delta-CT normalization and genomic instability test
	plot.py	creates all graphs
	statistics.py	runs all statistics
	regex_rename.py	function to allow flexible naming
application/template	all html interface files	creates the web form

Table S4: List of all the user inputs for the Auto-qPCR program and purpose of the expected user inputs. Section indicates the spot in the web app where the input box is located. User Input indicates the input box or options as they appear in the web app. Selections and Values indicates possible options for the user to select and the purpose of the input.

**Section heading**	**Input item**	**Description**
Select model	Click model	Choose the analysis model to run
Upload your data	Browse	Select your file(s), csv, txt, xml
File information	Yes or no	Choose yes if your file doesn't contain gene names or you want to filter out data from a second probe.
Normalization options	Endogenous control	Genes/targets for normalization
	Reference Sample	Control sample for ΔΔCT or instability tests
Options for removing technical replicates	CT-SD cut-off	The threshold for which the standard deviation is above and outliers from technical replicates will be removed. Default = 0.3
	Max proportion	The proportion of replicates that can be removed. Default = 0.5. With 0.5, if there are 3 replicates, only 1 can be removed
	Preserve highly variable replicates	If set to yes, a second condition is added before a replicate is removed. The difference between the mean and median must be greater than 10 % of the mean
Visualization Options	Target order	Genes are entered in the order they will appear on the graph
	Sample order	Sample names are entered in the order they will appear on the graph
Statists	Yes or no	More selections appear when yes is selected

Table S5: Description of the statistical tests using each possible selection criteria. The number of groups to compare, '#G' indicates the number of conditions to compare with the variables. The number of variables, '#Var' indicates the number of experimental conditions to compare. The distribution of the data determines if a parametric test will be used, for normally distributed data, or a non-parametric test will be used by the software. 'Measure' indicates if the data was collected on independent samples or on the same samples at different time points. 'Test' indicates the name of the test used by the software based on the user's sections from the other four criteria. Auto-qPCR always uses the same post-hoc test except when only two groups are being compared and no post-hoc test is performed.

# G	# V	Distribution	Measure	Test	Posthoc
2	1	parametric (normal)	Independent	student t-test two tailed, un-paired	none
2	1	parametric (normal)	Repeated measures (dependent)	student t-test two tailed, paired	none
2	1	non-parametric	Independent	Wilcoxon test	none
2	1	non-parametric	Repeated measures (dependent)	Mann-Whitney U test	none
> 2	1	parametric (normal)	Independent	one-way ANOVA	pairwise t-tests with FDR correction
> 2	1	parametric (normal)	Repeated measures (dependent)	one-way ANOVA	pairwise t-tests with FDR correction
> 2	1	non-parametric	Independent	Kruskal-Wallis test	pairwise t-tests with FDR correction
> 2	1	non-parametric	Repeated measures (dependent)	Friedman test	pairwise t-tests with FDR correction
> 2	2	parametric (normal)	Independent	two-way ANOVA	pairwise t-tests with FDR correction
> 2	2	parametric (normal)	Repeated measures (dependent)	two-way ANOVA	pairwise t-tests with FDR correction, for conditions 1,2 and the interaction

Table S6: Results of Auto-qPCR summary output found in summary_data.csv. The DNA region is indicated in Target Name, cell lines are indicated in Sample Name, Indel indicates if there is a duplication or deletion event calculated by the web app, Rep is the number of technical replicates included for analysis, RQ is the relative quantification, Std is the standard deviation and SEM is the standard error of the mean. RQ values from the technical replicates.

Target Name	Sample Name	Indel	Rep	RQ	Std	SEM
CHR1	GM25953	Normal	3	0.958	0.028	0.016
CHR1	GM25975	Normal	3	1.009	0.036	0.021
CHR1	GM25974	Normal	3	1.026	0.011	0.006
CHR1	GM25952	Normal	3	0.962	0.058	0.033
CHR1	Normal	Normal	3	1.000	0.032	0.019
CHR4	GM25953	Normal	3	1.000	0.006	0.003
CHR4	GM25975	Normal	3	1.000	0.012	0.007
CHR4	GM25974	Normal	3	1.000	0.016	0.009
CHR4	GM25952	Normal	3	1.000	0.024	0.014
CHR4	Normal	Normal	3	1.000	0.017	0.010
CHR8	GM25953	Normal	3	1.026	0.035	0.020
CHR8	GM25975	Normal	3	1.027	0.053	0.031
CHR8	GM25974	Normal	3	1.102	0.006	0.003
CHR8	GM25952	Normal	3	1.007	0.028	0.016
CHR8	Normal	Normal	3	1.000	0.009	0.005
CHR10	GM25953	Normal	3	0.913	0.040	0.023
CHR10	GM25975	Normal	3	0.998	0.024	0.014
CHR10	GM25974	Normal	3	0.976	0.044	0.026
CHR10	GM25952	Normal	3	0.979	0.061	0.035
CHR10	Normal	Normal	3	1.000	0.008	0.005
CHR12	GM25953	Normal	3	0.935	0.038	0.022
CHR12	GM25975	Normal	3	1.094	0.005	0.003
CHR12	GM25974	Normal	3	1.140	0.023	0.013
CHR12	GM25952	Normal	3	1.080	0.053	0.031
CHR12	Normal	Normal	3	1.000	0.012	0.007
CHR17	GM25953	Normal	3	0.921	0.054	0.031
CHR17	GM25975	Normal	3	1.061	0.061	0.035
CHR17	GM25974	Normal	3	1.220	0.041	0.024
CHR17	GM25952	Normal	3	1.088	0.202	0.116
CHR17	Normal	Normal	3	1.001	0.049	0.028
CHR18	GM25953	Normal	3	0.938	0.021	0.012
CHR18	GM25975	Normal	3	0.991	0.028	0.016
CHR18	GM25974	Normal	3	0.972	0.032	0.019
CHR18	GM25952	Normal	3	0.988	0.010	0.006
CHR18	Normal	Normal	3	1.000	0.015	0.009
CHR20	GM25953	Normal	3	0.992	0.045	0.026
CHR20	GM25975	Normal	3	1.104	0.007	0.004
CHR20	GM25974	Normal	3	0.927	0.025	0.014
CHR20	GM25952	Normal	3	0.874	0.021	0.012
CHR20	Normal	Normal	3	1.000	0.037	0.021
CHRX	GM25953	Normal	3	0.963	0.030	0.018
CHRX	GM25975	Normal	3	0.931	0.019	0.011
CHRX	GM25974	Normal	3	0.975	0.007	0.004
CHRX	GM25952	Normal	3	0.985	0.069	0.040
CHRX	Normal	Normal	3	1.000	0.027	0.016

Table S7: Statistical results for the absolute quantification found in file ANOVA_results.csv. Target Name indicates the genes compared, DF: degrees of freedom, F is the statistic to determine the p-value, MS: mean squares, SS: sums of squares, measure indicates if the tests were dependent measures for example, in a time course, where cell lines were matched across samples. Dist indicates the distribution is normal (parametric).

Target Name	DF	F	MS	SS	p-value	p-value corrected	Measure	Dist
GAPDH	3	5.491	0.046	0.137	0.00951	0.04753	dependent	parametric
ACTB	3	6.958	0.038	0.115	0.00372	0.01859	dependent	parametric
KCNJ6	3	22.923	20.729	62.188	0.00001	0.00004	dependent	parametric
SYP	3	114.917	58.478	175.433	0.00000	0.00000	dependent	parametric
GRIA1	3	11.24	10.081	30.243	0.0004	0.00201	dependent	parametric

Table S8: Post-hoc results from the statistical analysis of the absolute quantification from the one-way ANOVA. These results are found in file Posthoc_result.csv. The comparisons between individual stages for each gene is show. Target Name indicates the gene of interest. **A and B** show the two groups being compared. DF: degrees of freedom, p-value correct is the value corrected for multiple comparisons, p-value before correction for a paired t-test. Parametric, True means a normal distribution was selected.

Target Name	A	B	DF	p-value corrected	p-value	Paired	Parametric
KCNJ6	IPSC	NPC	5	0.85667	0.73431	TRUE	TRUE
KCNJ6	IPSC	DA4W	5	0.00845	0.00282	TRUE	TRUE
KCNJ6	IPSC	DA6W	5	0.00845	0.00253	TRUE	TRUE
KCNJ6	NPC	DA4W	5	0.01157	0.00705	TRUE	TRUE
KCNJ6	NPC	DA6W	5	0.01157	0.00771	TRUE	TRUE
KCNJ6	DA4W	DA6W	5	0.85667	0.85667	TRUE	TRUE
SYP	IPSC	NPC	5	0.18543	0.171	TRUE	TRUE
SYP	IPSC	DA4W	5	0.0001	0.00002	TRUE	TRUE
SYP	IPSC	DA6W	5	0.00018	0.00009	TRUE	TRUE
SYP	NPC	DA4W	5	0.00018	0.00012	TRUE	TRUE
SYP	NPC	DA6W	5	0.00018	0.00011	TRUE	TRUE
SYP	DA4W	DA6W	5	0.18543	0.18543	TRUE	TRUE
GRIA1	IPSC	NPC	5	0.06779	0.05649	TRUE	TRUE
GRIA1	IPSC	DA4W	5	0.03575	0.01192	TRUE	TRUE
GRIA1	IPSC	DA6W	5	0.03575	0.01137	TRUE	TRUE
GRIA1	NPC	DA4W	5	0.06779	0.03449	TRUE	TRUE
GRIA1	NPC	DA6W	5	0.06779	0.0519	TRUE	TRUE
GRIA1	DA4W	DA6W	5	0.35174	0.35174	TRUE	TRUE

Table S9: Example of output from the relative delta-CT analysis from the file clean_data.csv showing the top 10 rows of data. Target Name indicates the gene analyzed, Sample Name indicates the cell line, rq is the relative quantification for each replicate, rq-mean is the mean value of the replicates, rqSD is the standard deviation of the replicates, rqSEM is the standard error of the replicates, Outliers indicates if each outlier is a replicate, Group indicates the group used for statistics for the summary data.

Target Name	Sample Name	rq	rqMean	rqSD	rqSEM	Outliers	Group
PAX6	AIW002-2	0.0187	0.0223	0.0032	0.0018	FALSE	D0
PAX6	AIW002-2	0.0248	0.0223	0.0032	0.0018	FALSE	D0
PAX6	AIW002-2	0.0235	0.0223	0.0032	0.0018	FALSE	D0
PAX6	AIW002-2	0.0072	0.0073	0.0004	0.0002	FALSE	D7
PAX6	AIW002-2	0.0069	0.0073	0.0004	0.0002	FALSE	D7
PAX6	AIW002-2	0.0077	0.0073	0.0004	0.0002	FALSE	D7
PAX6	KYOU	0.1261	0.1193	0.0065	0.0038	FALSE	D0
PAX6	KYOU	0.1131	0.1193	0.0065	0.0038	FALSE	D0
PAX6	KYOU	0.1187	0.1193	0.0065	0.0038	FALSE	D0
PAX6	KYOU	0.0202	0.0210	0.0007	0.0004	FALSE	D7

Table S10: Statistical results from the relative quantification comparing the delta-CT and delta-delta-CT using student t-tests. Target Name indicates the gene being compared, DF: degrees of freedom, tail; two tail t-test, paired FALSE indicated an unpaired t-test. Model indicates if the delta-CT or the delta-delta-CT method was used. Effect size is the difference between the means divided by the SD. Power is the probability of a type I error minus the probability of a type II error. The Bayes factors is ration between the probability of the null hypothesis and the alternative hypothesis being true. Values < 1 support the null hypothesis and values > 1 support the alternative hypothesis. The values seen here are only considered trends, a Bayes factor between 3-10 is considered moderate, 10-100 strong, >100 extreme support for the alternative hypothesis.

**Target Name**	**DF**	**T**	**tail**	**paired**	**p-value**	**model**	**effect size**	**power**	**Bayes factor**
PAX6	1	1.361	two-sided	FALSE	0.40342	delta CT	1.449	0.129	0.847
CAMK2A	1	-3.277	two-sided	FALSE	0.18855	delta CT	1.405	0.125	1.359
GRIN1	1	-3.744	two-sided	FALSE	0.16616	delta CT	1.836	0.162	1.454
PAX6	1	1.361	two-sided	FALSE	0.40342	delta delta CT	1.449	0.129	0.847
CAMK2A	1	-3.277	two-sided	FALSE	0.18855	delta delta CT	1.405	0.125	1.359
GRIN1	1	-3.744	two-sided	FALSE	0.16616	delta delta CT	1.836	0.162	1.454

Table S11: Manual processing compared to Auto-qPCR processing with a range of cut-off values for std to exclude replicates, with or without preserving highly variable outliers. Calculations are all using the absolute model to quantify NRXN3 expression with and without cocaine treatment in three brain regions. Values that differ across processing conditions are highlighted in bold. **Left**, the sample information for Region, Treatment and code name of each mouse (biological replicate) are listed. The processing methods, Manual or Auto-qPCR, are labelled. The CT-SD cut-off is the value for which std exceeded for outliers to be moved. The settings for preserving highly variable technical if the ration of mean-media/media is less than 0.1 is indicated by 'yes'. RNA indicates the RNA quantification values.

			Manual	Auto-qPCR				
**Preserve high variation replicates**			yes	no	no	no	yes	yes
**CT-SD cut-off**			0.29	0.3	0.275	0.2	0.3	0.275
**Region**	**Treatment**	**Mouse**	**RNA**	**RNA**	**RNA**	**RNA**	**RNA**	**RNA**
STN	Saline	B4bis	0.2564	0.2564	0.2564	**0.2817**	0.2564	0.2564
STN	Saline	B6	0.1933	0.1933	0.1933	0.1933	0.1933	0.1933
STN	Saline	R6	0.3290	0.3290	0.3290	**0.3055**	0.3290	0.3290
STN	Saline	V3	0.2845	0.2845	0.2845	**0.3357**	0.2845	0.2845
STN	Saline	V4	0.3259	0.3259	0.3259	0.3259	0.3259	0.3259
STN	Cocaine	R5Bis	0.4570	0.4570	0.4570	0.4570	**0.4116**	**0.4116**
STN	Cocaine	R6bis	0.1708	0.1708	0.1708	0.1708	0.1708	0.1708
STN	Cocaine	R8bis	0.4253	0.4253	0.4253	0.4253	0.4253	0.4253
STN	Cocaine	V2	0.2538	**0.1659**	**0.1659**	**0.1659**	**0.1987**	**0.1987**
STN	Cocaine	V8	0.1818	0.1818	0.1818	0.1818	0.1818	0.1818
GP	Saline	B4bis	0.2541	0.2541	0.2541	0.2541	0.2541	0.2541
GP	Saline	R6	0.7107	0.7107	0.7107	0.7107	0.7107	0.7107
GP	Saline	V3	0.4125	0.4125	0.4125	0.4125	0.4125	0.4125
GP	Saline	V4	0.2991	0.2991	0.2991	0.2991	0.2991	0.2991
GP	Cocaine	R5Bis	0.5021	0.5021	0.5021	0.5021	**0.4988**	**0.4988**
GP	Cocaine	R6bis	0.9500	0.9500	0.9500	0.9500	0.9500	0.9500
GP	Cocaine	R8bis	1.0169	1.0169	1.0169	**0.9455**	1.0169	1.0169
GP	Cocaine	V2	0.8538	0.8538	0.8538	**0.7797**	0.8538	0.8538
GP	Cocaine	V8	0.9486	0.9486	0.9486	0.9486	0.9486	0.9486
SN	Saline	B4bis	0.7854	**0.8745**	0.7854	0.7854	**0.7317**	**0.7317**
SN	Saline	R6	0.8751	**1.0784**	**1.0784**	**1.0784**	0.8751	0.8751
SN	Saline	V3	1.3306	1.3306	1.3306	1.3306	**1.1553**	**1.1553**
SN	Saline	V4	1.0575	1.0575	1.0575	1.0575	**0.8940**	**0.8940**
SN	Cocaine	R5Bis	1.2379	1.2379	1.2379	1.2379	1.2379	1.2379
SN	Cocaine	R6bis	1.0016	**1.1607**	**1.1607**	**1.2982**	1.0016	1.0016
SN	Cocaine	R8bis	0.4393	0.4393	0.4393	0.4393	0.4393	0.4393
SN	Cocaine	V2	1.0196	1.0196	1.0196	**1.0971**	1.0196	1.0196
SN	Cocaine	V8	2.2979	2.2979	2.2979	2.2979	2.2979	2.2979

Table S12: Comparison of variance between manual processing and Auto-qPCR. The variance between RNA quantity values calculated manually or with Auto-qPCR were calculated between each mean value found in table S11. For each brain region the sum of the variance was calculated. The same comparison was performed between manual processing and Auto-qPCR with the standard cut-off of 0.3 and the standard cut-off together plus the preserve extreme values option.

**Region**	**Cut-off 0.3**	**Cut-off 0.3 + Preserve**
STN	0.004	0.003
GP	0.000	0.000
SN	0.037	0.030
All regions	0.037	0.033

Table S13: Addition of group columns used for statistical analysis for one-way and two-way ANVOAs. Treatment (Control, Cocaine), Region (STN,GP,SN) and both together, T_R (STN_Control, STN_Cocaine, GP_Control, GP_Cocaine, SN_Control, SN_Cocaine). Each group column was used for separate one-way ANOVAs. The 'Treatment' and 'Region' column were used in the two way ANOVA. For visualization the first 15 rows with sample data are shown from the full spreadsheet.

Well /set	Dye	Content	Description	Efficiency	C(t)	ng	T_R	Treatment	Region
B1	FAM	Sample	B4bisNST	50.57%	30.71	0.128	STN_Control	Control	STN
B1	TMR	Sample	B4bisNST	N/A	N/A		STN_Control	Control	STN
B2	FAM	Sample	B4bisNST	45.00%	30.29	0.170	STN_Control	Control	STN
B2	TMR	Sample	B4bisNST	N/A	N/A		STN_Control	Control	STN
B3	FAM	Sample	B4bisNST	46.18%	30.2	0.181	STN_Control	Control	STN
B3	TMR	Sample	B4bisNST	N/A	N/A		STN_Control	Control	STN
B4	FAM	Sample	B4bisGP	32.11%	26.6	2.053	GP_Control	Control	GP
B4	TMR	Sample	B4bisGP	N/A	N/A		GP_Control	Control	GP
B5	FAM	Sample	B4bisGP	45.30%	28.43	0.598	GP_Control	Control	GP
B5	TMR	Sample	B4bisGP	N/A	N/A		GP_Control	Control	GP
B6	FAM	Sample	B4bisGP	47.44%	28.18	0.708	GP_Control	Control	GP
B6	TMR	Sample	B4bisGP	N/A	N/A		GP_Control	Control	GP
B7	FAM	Sample	B4bisSN	47.60%	27.56	1.073	SN_Control	Control	SN
B7	TMR	Sample	B4bisSN	N/A	N/A		SN_Control	Control	SN

Table S14: Results of the statistical analysis of control vs. cocaine treatment for all brain regions. The results of an unpaired, two tailed students t-test performed in Auto-qPCR using the statistic selections. The table is found in the file 'ttest_results.csv'.

Target Name	DF	T	tail	paired	p-val
B2M	18.54	0	two-sided	FALSE	1.000
NRXN3	22.74	-1.555	two-sided	FALSE	0.134

Table S15: Posthoc results after one-way ANOVA comparing brain regions. Control and cocaine treatment samples were pooled together. Target name indicates the gene tested. **A and B** indicate the two regions being compared. DF: degree of freedom.

Target Name	A	B	DF	p-value corrected	p-value	correction method
B2M	STN	GP	9	1.0000	1.0000	fdr_bh
B2M	STN	SN	12	1.0000	1.0000	fdr_bh
B2M	GP	SN	16	1.0000	1.0000	fdr_bh
NRXN3	STN	GP	9	0.0071	0.0047	fdr_bh
NRXN3	STN	SN	8	0.0048	0.0016	fdr_bh
NRXN3	GP	SN	16	0.0549	0.0549	fdr_bh

Table S16: One-way ANOVA and posthoc test comparing groups of brain region and treatment. The ANOVA results are shown for both B2M and NRXN3 for the overall effect of treatment and brain region together (One-way ANOVA. The post-hoc tests for the relevant comparisons are shown for each brain region with and without cocaine treatment (post-hoc).

Target Name	Comparison	DF	p-value corrected	p-value	Test
B2M	Treatment and region	5	0.3885	0.7771	One-way ANOVA
NRXN3	Treatment and region	5	0.0006	0.0011	One-way ANOVA
NRXN3	STN_Control vs STN_Cocaine	8	0.9977	0.9977	post-hoc
NRXN3	GP_Control vs GP_Cocaine	7	0.0176	0.0334	post-hoc
NRXN3	SN_Control vs SN_Cocaine	5	0.4127	0.4762	post-hoc

Table S17: Two-way ANOVA and posthoc tests comparing brain region, treatment and interaction. The relevant information was selected from the output files 'ANOVA_results.csv' and 'Posthoc_results.csv'. The 2-way ANOVA results are shown for NRXN3 for the overall effect of brain region (Group1), treatment (Group2) and the interaction effect of region and treatment (Group1*Group2) (upper table). The post-hoc tests for the relevant comparisons are shown for each brain region with and without cocaine treatment for each brain region indicated under contrast. The 2-way ANOVA results are shown on top and the post-hoc multiple t-test comparisons are shown on the bottom, indicated in the Test column

Target Name	Contrast	DF	p-value corrected	p-value	Test
NRXN3	Group1: Region	2	0.0004	0.0001	ANOVA
NRXN3	Group2: Treatment	1	0.2265	0.0755	ANOVA
NRXN3	Group1 * Group2	2	1.0000	0.3513	ANOVA
NRXN3	all: Control vs Cocaine	23	NA	0.1337	post-hoc
NRXN3	STN: Control vs Cocaine	8	0.9977	0.9977	post-hoc
NRXN3	GP: Control vs Cocaine	7	0.0529	0.0176	post-hoc
NRXN3	SN: Control vs Cocaine	5	0.6190	0.4127	post-hoc

Table S18: Comparison of Auto-qPCR with similar open-source analysis programs.

Feature	Auto-q-PCR	SATQPCR [1]	PIPE-T [2]	Q-PCR [3]	Do my qPCR calculation [4]
Input type	CQ	CQ	rawCt.Cq Values	raw	CQ
Paste input into web	no	no	no	no	yes
Upload files	txt,csv,xml	txt	txt	RDML, csv	tsv, xlsx, ods
Files can be uploaded without reformatting	yes	no	yes	yes	no
CQ calculation	no	no	no	yes	no
Normalization and RQ<br>Absolute model	yes	no	no	no	no
Normalization and RQ<br>Relative dCT model	yes	yes	yes	yes	no
Normalization and RQ<br>Relative ddCT model	yes	yes	yes	yes	yes
User selections for removal of technical replicates	yes	no	yes	yes	no
Can account for PCR efficiency	no	yes	no	yes	yes
Allows multiple genes for normalization	yes	yes	yes	yes	yes

Output summary values	csv	txt	txt	xls, csv	xlsx
Output all technical replicates	csv	txt	txt	no	xlsx
Output graphs	png	jpg	png	svg, png	xlsx
statistics	Multiple tests	T-test or Anova with Tukey test	T-test<br>Two sample Wilcoxon test<br>Rank product	Anova, t-test, permutation test	t-test
Web based	Yes	Yes	No	Yes	yes
Requires login	No	No	No	Yes	no
Release date	2021	2019	2019	Original publication:<br>2009<br>2013 update	2019
Reference	This paper	1	2	3	4

References:

1 Rancurel, C., van Tran, T., Elie, C. & Hilliou, F. SATQPCR: Website for statistical analysis of real-time quantitative PCR data. *Mol Cell Probes* **46**, 101418, doi:10.1016/j.mcp.2019.07.001 (2019).

2 Zanardi, N. *et al.* PIPE-T: a new Galaxy tool for the analysis of RT-qPCR expression data. *Sci Rep* **9**, 17550, doi:10.1038/s41598-019-53155-9 (2019).

3 Pabinger, S. *et al.* QPCR: Application for real-time PCR data management and analysis. *BMC Bioinformatics* **10**, 268, doi:10.1186/1471-2105-10-268 (2009).

4 Tournayre, J., Reichstadt, M., Parry, L., Fafournoux, P. & Jousse, C. "Do my qPCR calculation", a web tool. *Bioinformation* **15**, 369-372, doi:10.6026/97320630015369 (2019).

File S1: Required packages and versions used for running Auto-qPCR. These packages are found in the document ‘requirments.txt’ and are installed in the virtual environment using python3 and pip.

blinker==1.4
certifi==2021.5.30
chardet==4.0.0
click==8.0.1
cycler==0.10.0
Flask==2.0.1
Flask-Mail==0.9.1
idna==3.2
itsdangerous==2.0.1
Jinja2==3.0.1
joblib==1.0.1
kiwisolver==1.3.1
littleutils==0.2.2
MarkupSafe==2.0.1
matplotlib==3.3.4
numpy==1.19.5
outdated==0.2.1
pandas==1.1.5
pandas-flavor==0.2.0
patsy==0.5.1
Pillow==8.3.2
pingouin==0.3.7
pyparsing==2.4.7
python-dateutil==2.8.2
pytz==2021.1
requests==2.26.0
six==1.16.0
statsmodels==0.12.2
tabulate==0.8.9
threadpoolctl==2.2.0
urllib3==1.26.6
Werkzeug==2.0.1
xarray==0.16.2
